# Comparing and contrasting different herbal products intended for the management of obesity approved in the Palestinian markets

**DOI:** 10.1186/s12906-022-03830-8

**Published:** 2023-01-05

**Authors:** Ahmad M. Eid, Linda Issa, Karmah Kamal, Omran Hosheya, Hla Sara, Sally Abed Alkader

**Affiliations:** Department of Pharmacy, Faculty of Medicine and Health Sciences, An-Najah National University, P.O. Box 7, Nablus, Palestine

**Keywords:** Obesity, Anti-lipase, Antioxidant, Anti-amylase

## Abstract

**Background:**

The use of conventional medical therapies has proven to have many setbacks and safety concerns that need further improvement. However, herbal medicine has been used for over 2000 years, and many studies have proven the use of herbs to be effective and safe. This article discussed the efficacy of different herbal products used in the management of obesity. To evaluate the efficacy of seven herbal-based weight loss products currently available on the Palestinian market, using in vitro assays to screen for antioxidants, anti-amylase, and anti-lipase effects for each product.

**Method:**

Pancreatic lipase and salivary amylase inhibitory activities, as well as antioxidant analysis, were tested in vitro on a variety of herbal products. Then the IC_50_ was measured for each test.

**Results:**

The anti-lipase assay results, IC_50_ values in (μg/mL) of each of the seven products (Product A, product B, product C, product D, product E, product F, and product G) were 114.78, 532.1, 60.18, 53.33, 244.9, 38.9, and 48.97, respectively. The IC_50_ value for orlistat (Reference) was 12.3 μg/ml. On the other hand, the IC_50_ value for alpha amylase inhibition of the seven products (Product A, product B, product C, product D, product E, product F, and product F) were 345.93, 13,803.84 (Inactive), 73.79, 130.91, 165.95, 28.18, and 33.11 μg/ml respectively, while acarbose (Reference) was 23.38 μg/ml. The antioxidant activity (IC_50_ values) for the seven products (Product A, product B, product C, product D, product E, product F, and product F) were 1258.92, 707.94, 79.43, 186.20, 164.81, 17.53, and 10.47 μg/ml respectively. While the IC_50_ value for Trolox was 2.70 μg/ml.

**Conclusion:**

It can be concluded that the seven products showed varied anti-lipase, anti-amylase, and antioxidant effects. However, products F and G showed superiority in all categories.

## Background

Obesity, or corpulence, is one of the most serious global public health challenges of the twenty-first century [1–3]. It’s an intricate disease that involves an uncontrolled amount of fat [4]. This medical issue increases the risk of many health problems that are considered the major causes of death and morbidity [5, 6]. Lately, the world of the pharmaceutical industry has been taking an interest in obesity and all its comorbid diseases, including cardiovascular and cerebrovascular, all of which are considered serious life threats that require immediate, effective, and safe therapies to be managed [7, 8].

Commonly, corpulence is a result of genetic, behavioral, physiological, and environmental factors combined with an individual’s diet and physical activity [9, 10]. Generally, the diet would include a huge number of calories from sugary, and fatty meals that could only be burned off through intense exercise [11]. The remaining calories will be stored in the body in the form of fat. People suffering from obesity are more likely to develop heart disease and strokes, type 2 diabetes, digestive problems, sleep apnea, osteoarthritis, severe COVID-19 symptoms, and certain cancers [12–14]. These complications are the leading causes of poor health and death worldwide. Obesity also diminishes the quality of life. It causes depression and disability, alongside additional psychological factors like less successful work, discomfort, and ignominy [15].

The management of obesity is still a paradox. It is aimed at altering the diet and increasing physical activity through nutritional counseling [16, 17]. However, patient compliance is frequently limited, therefore, pharmacological treatment was the next alternative [18]. Nevertheless, research showed that the conventional therapy of obesity with orlistat, sibutramine, and dexfenfluramine is effective when such drugs are used to control the accumulation of fat in the white adipose tissue [19–21]. However, they have manifested serious side effects, including mental disorders, non-fatal myocardial infarctions or strokes, and depression, which restrict their use [19, 22].

The increasing necessity for another form of therapy prompted the development of research into the use of herbal medicine and natural products [23, 24]. These drugs are now considered an alternative anti-obesity therapy with superior potency and safety [25]. One of these herbs that is so frequently and traditionally used for weight-loss is green tea [26]. The combination of catechins and caffeine in green tea is responsible for its weight loss mechanism [27]. Catechins upregulate hepatic enzymes to stimulate fat oxidation while inhibiting catechol-O-methyltransferase. As a result, glucose uptake is significantly reduced while lipolysis is increased [28]. Caffeine, on the other hand, stimulates the sympathetic nervous system and increases uncoupling proteins, which cause energy expenditure and fat oxidation [29]. Green tea has been successfully proven to have weight loss effects as well as reduce fat absorption and inhibit lipase activity [30].

Among these herbs, as well, is *Hoodia Gordonii*. This plant works by inhibiting the appetite and thirst, giving a feeling of early satiety. Its chemical constituent screen showed it to contain oxypregnane steroidal glycoside P57AS3, which acts in a glucose memetic manner that reduces food interest and appetite and inhibits hunger signals. It is often combined with *Momordica charantia*, also known as bitter melon. The triterpenoids, saponins, phenolics, and linolenic acids making up the bitter melon are largely responsible for its anti-obesity effect. They all work by inhibiting fat production and stimulating glucose consumption.

*Taraxacum officinale* is another widely used herb for weight loss [31]. It works by inhibiting pancreatic and gastric lipases, thus inhibiting fat absorption and digestion of triglycerides [32, 33]. Furthermore, it works as an antioxidant responsible for fat breakdown and blocking fat accumulation [33, 34]. This herb works in the same way orlistat does and is believed to replace it in the future because of the gastrointestinal side effects of orlistat, which limited its use [31, 35].

Finally, orlistat (Xenical) is a pharmacological agent promoting weight loss in obese subjects via inhibition of gastric and pancreatic lipase, an enzyme that is crucial for the digestion of long-chain triglycerides [36]. Herbs and spices are generally considered safe and have been proven to be effective against various human ailments [37, 38]. In recent years, many herbal combinations have been developed to meet the need for high quality, clinically evaluated, botanical body weight management dietary ingredients. For example, an in vitro screening program, established by Laila Nutraceuticals (Vijayawada, India), evaluated hundreds of herbal extracts for their ability to inhibit fat accumulation by adipocyte differentiation and potentiate fat breakdown within cells in mouse adipocytes [39]. Humerus3® was one of the previously recognized herbal formulations, and it may be particularly beneficial for individuals who have tried other therapies but found them ineffective in managing their appetite. A significant and long-lasting weight loss program is possible with Humerus3® [40]. These carefully chosen herbs contain natural effective compounds such as gingerenone, nigellicine, nigellidine, thymoquinone, thymol, carvacrol, alpha-pinene, myrcene, and transanthole [40].

This study aims to assess the anti-lipase, anti-amylase, and antioxidant activities of selected officially certified weight loss herbal products that are available in the Palestinian markets in order to confirm their efficacy and quality.

## Materials and methods

### Materials

In this research experiment, the following materials were acquired and used: Lipase from porcine pancreas (Sigma Aldrich) USA, P-nitrophenylbutyrate (PNPB), Tris-HCl buffer. and porcine pancreatic α-amylase from MP Biochemicals (Sigma Aldrich) USA. Dimethyl sulfoxide (DMSO) (Riedel-de-Haen, Germany). DPPH (2,2-diphenyl-1-picrylhydrazyl) (Sigma Aldrich, Germany), Trolox (Sigma Aldrich, Germany). and starch (Alzahra’a Palestine). For the sake of this experiment, the products purchased were called products (A®, B®, C®, D®, E®, F®, and G®), all of which are available in the Palestinian market and were obtained from a community pharmacy for in vitro assay. The constituents of each product are listed in Table 1.

**Table 1:** List of the products used in the study with their main ingredients

Products	Main ingredients as listed by the company
Product A®	Omtec 50 and Omtec XS.
Product B®	Litramine is a patented fiber complex (*Opuntia ficus-indica*) and vitamins A,D and E
Product C®	*Laurusnobilis, rheum*, *cymbopogoncitratus*, *mentaspecata*, *rosacania*, *camellia sinensis*, *matricariachamomilla*, hibiscus, and vitamin C.
Product D®	*Garcinia Cambogia* fruit extract
Product E®	*Hoodia Gordonii* extract
Product F®	Amino acids, vitamin B6, chromium, and *taraxacum officinale* extract
Product G®	Green tea extract, and phytosome complex

### Porcine pancreatic lipase inhibitory activity assay

A stock solution for each of the herbal products and orlistat, since orlistat is the only commercial drug on the market effective against pancreatic lipase, was prepared by dissolving the contents of each capsule in a 100 ml volumetric flask. Each solution underwent dilutions to give the following concentrations (50, 100, 200, 300, and 400 μg/ml). 0.2 ml of each diluted solution was added into a test tube. Immediately before use, one mg/ml stock solution of the porcine pancreatic lipase enzyme was prepared in a 10% DMSO solution. Subsequently, 0.7 ml of Tris-HCl buffer (pH 7.4, 0.1 M) was added, as well as 0.1 ml of pancreatic lipase enzyme. A blank tube holding 0.9 ml Tris-HCl and 0.1 ml pancreatic lipase enzyme without the inhibitor, as well as all previously discussed test tubes, were kept for 15 minutes at 37 °C as an incubation period. Another incubation period of 30 minutes takes place after the addition of 0.1 ml of PNPB (100 mM in acetonitrile) to all test tubes, including the blank, pancreatic lipase activity was evaluated by detecting the hydrolysis of PNPB to p-nitrophenol at 410 nm with a UV-visible spectrophotometer (Shimadzu-UV-1800, Kyoto, Japan) [41].

The following equation was later used to calculate the absolute inhibitory activity.

$$\textrm{Inhibition}\ \left(\%\right)=\frac{\left(\ \textrm{B}-\textrm{S}\right)}{\textrm{B}}X\ 100\%$$

Where S, refers to the sample absorbance and B to the blank absorbance.

### α-Amylase inhibitory assay

The amylase inhibitory action of the seven herbal products was assessed using the standard method [42], with a few modifications. Each of the herbal products was dissolved in a few milliliters of 10% DMSO, and later dissolved in solutions of 0.02 M sodium phosphate monobasic and sodium phosphate dibasic buffer involving 0.006 M sodium chloride (NaH2PO4 and Na2HPO4, NaCl, pH 6.9) to give a stock solution with a 1000 μg/ml concentration. Using 10% DMSO, dilutions were prepared with the following concentrations (10, 50, 70, 100, 500 μg/ml). Then, 0.2 ml of porcine pancreatic amylase enzyme solution (2 units/ml) was mixed with 0.2 ml of each herbal sample and left to incubate for 10 minutes at 30 °C. Meanwhile, 0.2 ml of freshly prepared 1% starch aqueous solution was incubated for 3 min before being mixed with each test tube.

To stop this reaction, 0.2 ml of DNSA color reagent was diluted with 5 ml of distilled water and then added. This mixture was allowed to boil for 10 minutes in a water bath at 90 °C before being allowed to cool to room temperature. The absorbance of the final mixture was assessed at 540 nm. The blank solution was made in the same steps discussed previously, with the addition of 0.2 ml of the previous buffer instead of the herbal samples. The acarbose was prepared using the same methods that were previously used for the herbal sample and was used as a reference.

The following equation was used to calculate the amylase inhibitory activity:

$$\upalpha -\textrm{amylase}\ \textrm{inhibitory}\%=\frac{\left(\textrm{AB}-\textrm{AT}\right)}{\textrm{AB}}X\ 100\%$$

AB: Recorded absorbance of the blank solution. AT: Recorded absorbance of the tested herbal sample solution.

### DPPH free radical-scavenging activity

Methanolic stock solution was prepared for each of the seven herbal products to achieve a concentration of 100 μg/ml. The stock solutions were then diluted to reach the concentrations of (5, 10, 20, 30, 50, 80, 100 μg/ml). Next, one ml of each diluted solution was added to a different test tube, and 1 ml of recently prepared the free radical DPPH (0.002 g/ml) methanolic solution was added to the mix. Subsequently, 2 ml of methanol and one ml DPPH (negative control) was added as well. In this assay, the blank solution was made of a combination in a 2:1 ratio of DPPH and methanol. Subsequently, prior solutions were left to incubate for 30 minutes at a room temperature of 25 °C in a dark setting. Finally, using the spectrophotometer, the optical densities of each solution were measured at 517 nm [42].

The following equation was used to calculate the percent of DPPH inhibited for all samples previously discussed, including the buffer:

$$\textrm{DPPH}\ \textrm{inhibition}\%=\frac{\left(\textrm{AB}-\textrm{AS}\right)}{\textrm{AS}}X\ 100\%$$

AB: Blank solution absorbance.

AS: Sample solution absorbance.

### Statistical analysis

The findings of this research were expressed as the mean ± standard deviation (SD). The IC_50_ (50% inhibitory concentration) was calculated using BioDataFit edition 1.02 [43].

## Results

### Porcine pancreatic lipase enzyme inhibitory

**Fig. 1:**
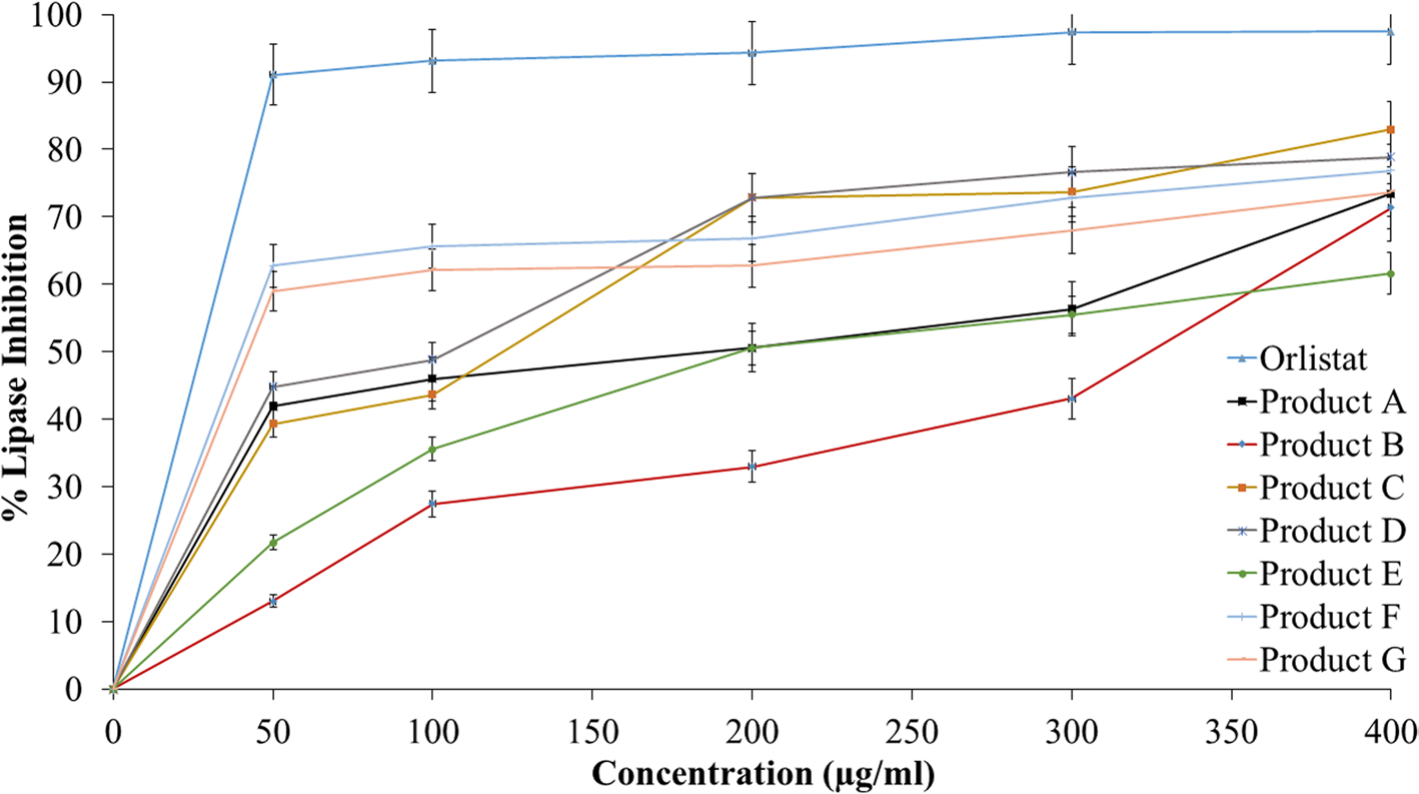
The inhibitory effect of orlistat and the products on lipase enzyme

**Fig. 2:**
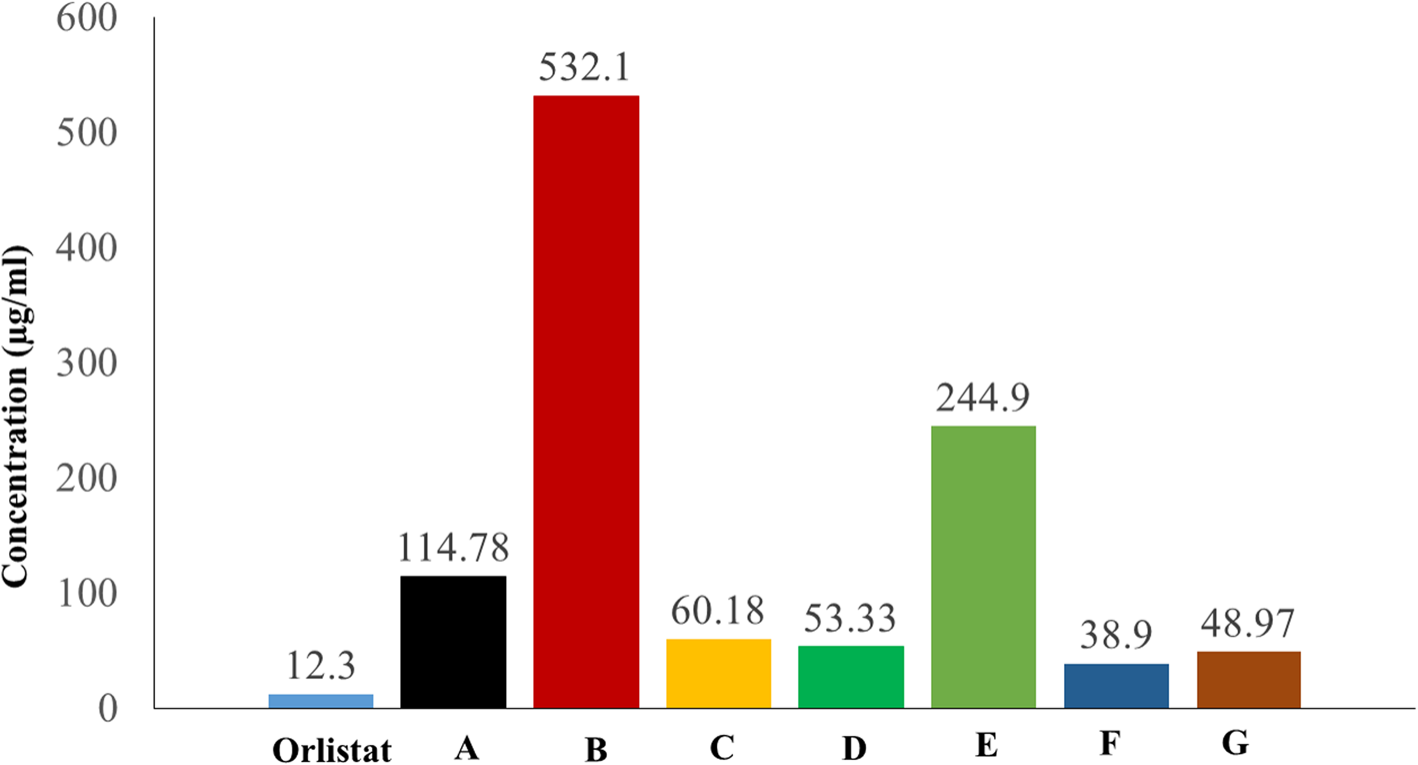
IC_50_ values in (μg/mL) for orlistat the tested products

The anti-lipase effect of all tested products was obtained and compared with the inhibitory effect of orlistat as shown in Fig. 1, the obtained results for the products ranged from strong to weak inhibition. The IC_50_ values of the seven products (A®, B®, C®, D®, E®, F®, and G®) were 114.78, 532.1, 60.18,53.33, 244.9, 38.9, and 48.97 μg/ml respectively, while the IC_50_ value of orlistat (The reference compound) was 12.3 μg/ml (Fig. 2). This showed that products C®, D®, F®, and G® had a great inhibitory effect on the porcine pancreatic lipase enzyme when compared to orlistat. Product F® showed superiority effect over other tested products. On the other hand, products A®, B®, and E® showed low effects, yet product B® showed the lowest of all.

### α-Amylase inhibitory results

**Fig. 3:**
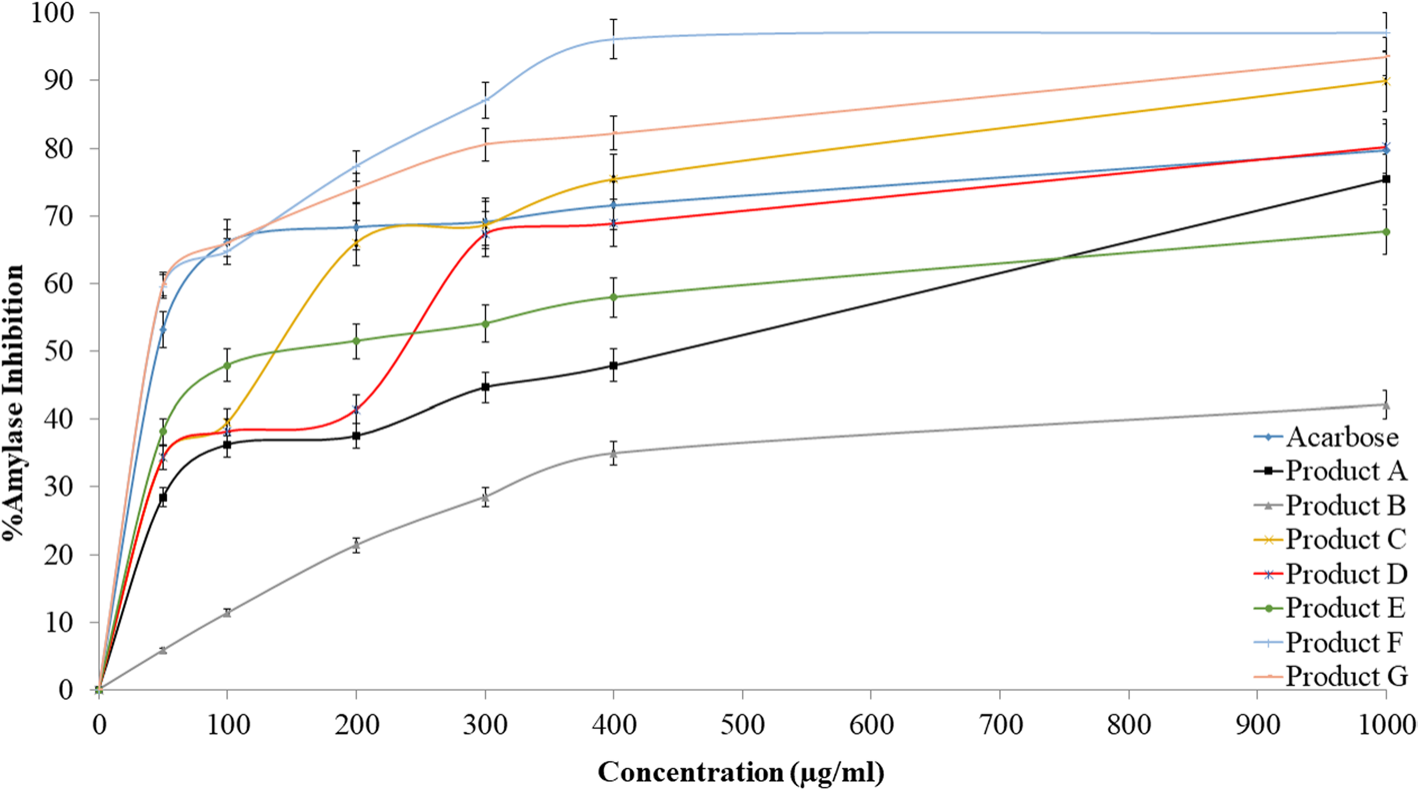
The inhibitory effect of acarbose and the tested products

**Fig. 4:**
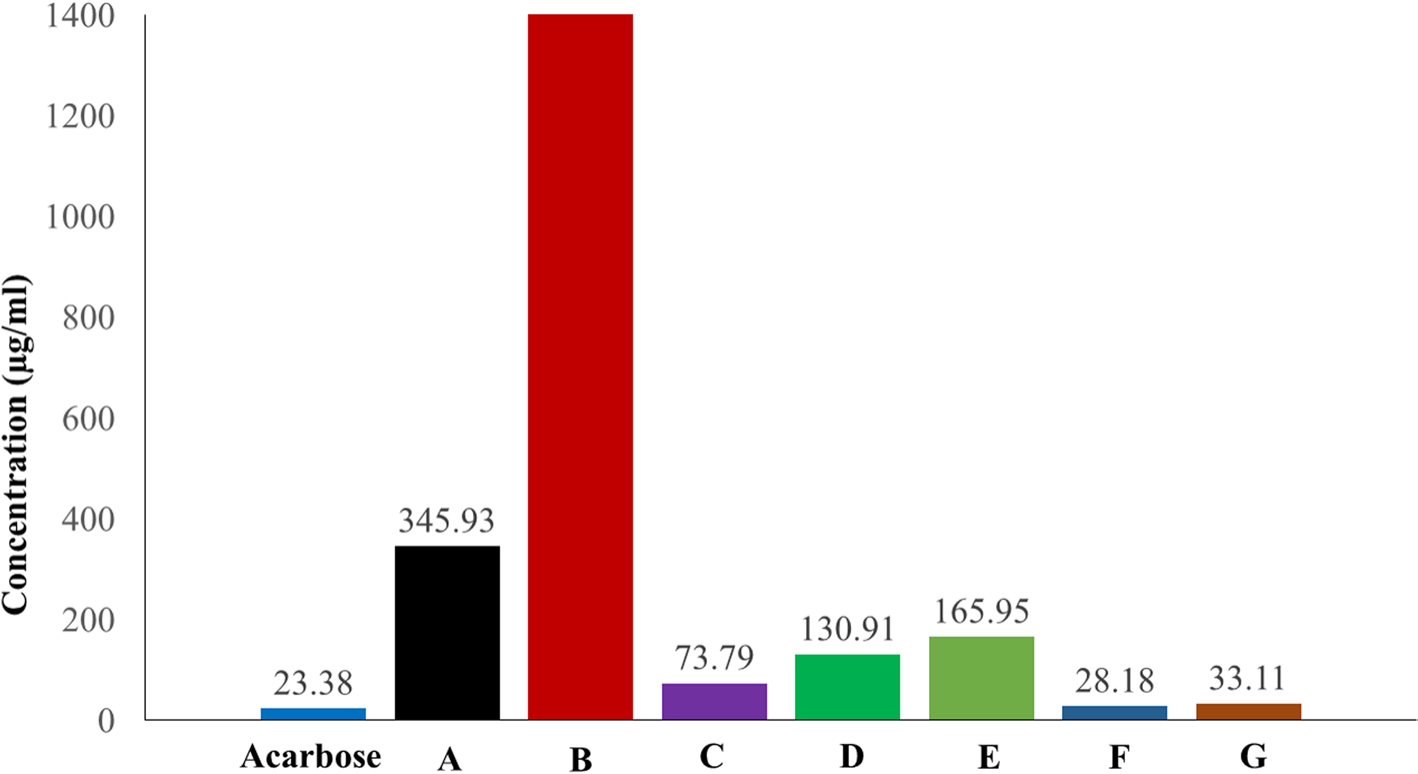
IC_50_ values in (μg/ml) for acarbose and the tested products

The inhibitory activity of the seven products was tested and compared to the positive control acarbose; the results are shown in Fig. 3. When compared to acarbose, products A®, B®, C®, D®, E®, F®, and G® had varying α-amylase inhibitory effects. The IC_50_ values for the seven products (A®, B®, C®, D®, E®, F®, and G®) were 345.93, 13,803.84 μg/mL (Inactive), 73.79, 130.91, 165.95, 28.18, and 33.11 μg/mL respectively (Fig. 4), while acarbose was 23.38 μg/ml. Products C®, F®, and G® showed the strongest inhibitory effects, in which product F® showed superiority over all. However, while products A®, D®, and E® showed low inhibition effects against α-amylase, product B® had no inhibitory effect.

### Antioxidant results

**Fig. 5:**
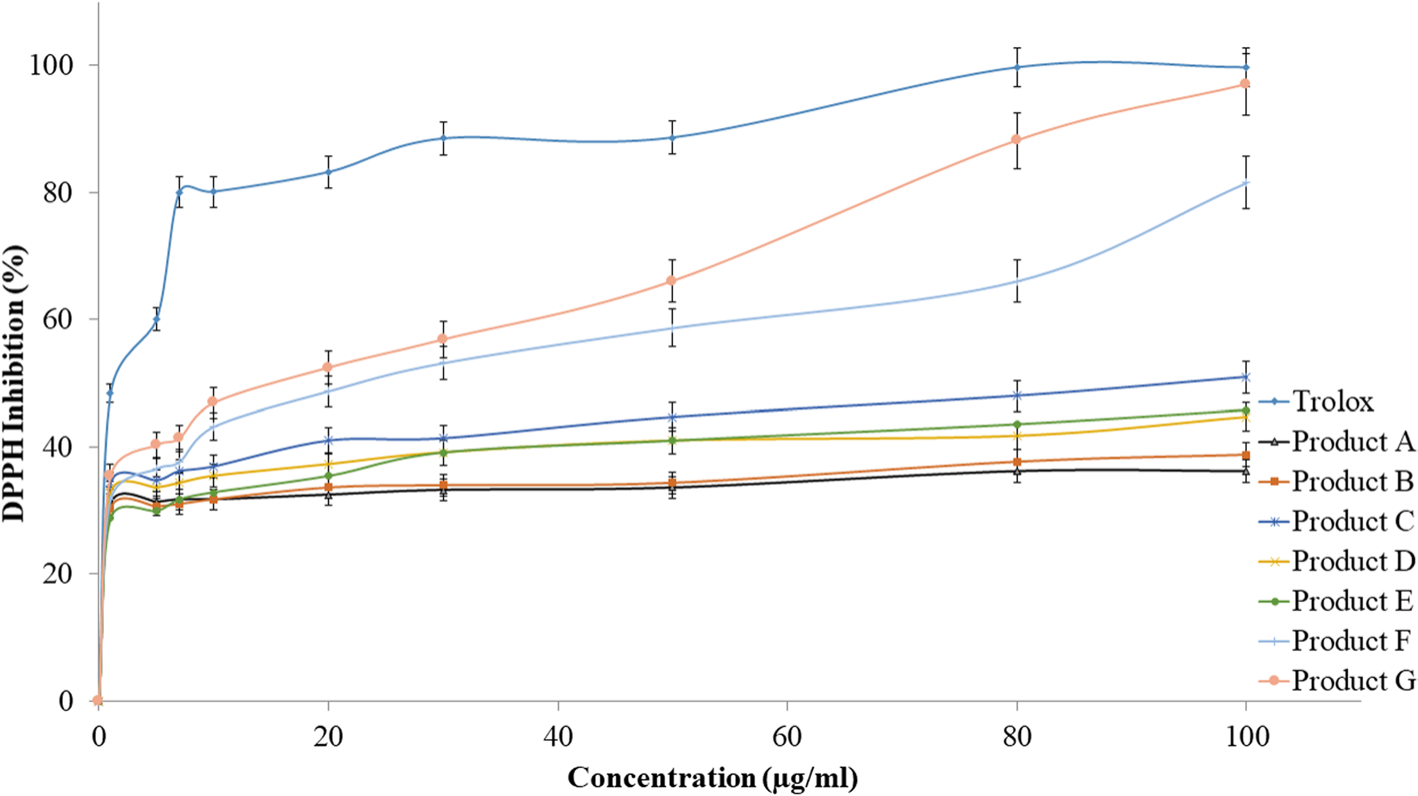
The percentages of DPPH inhibitory activity for the tested products and trolox

**Fig. 6:**
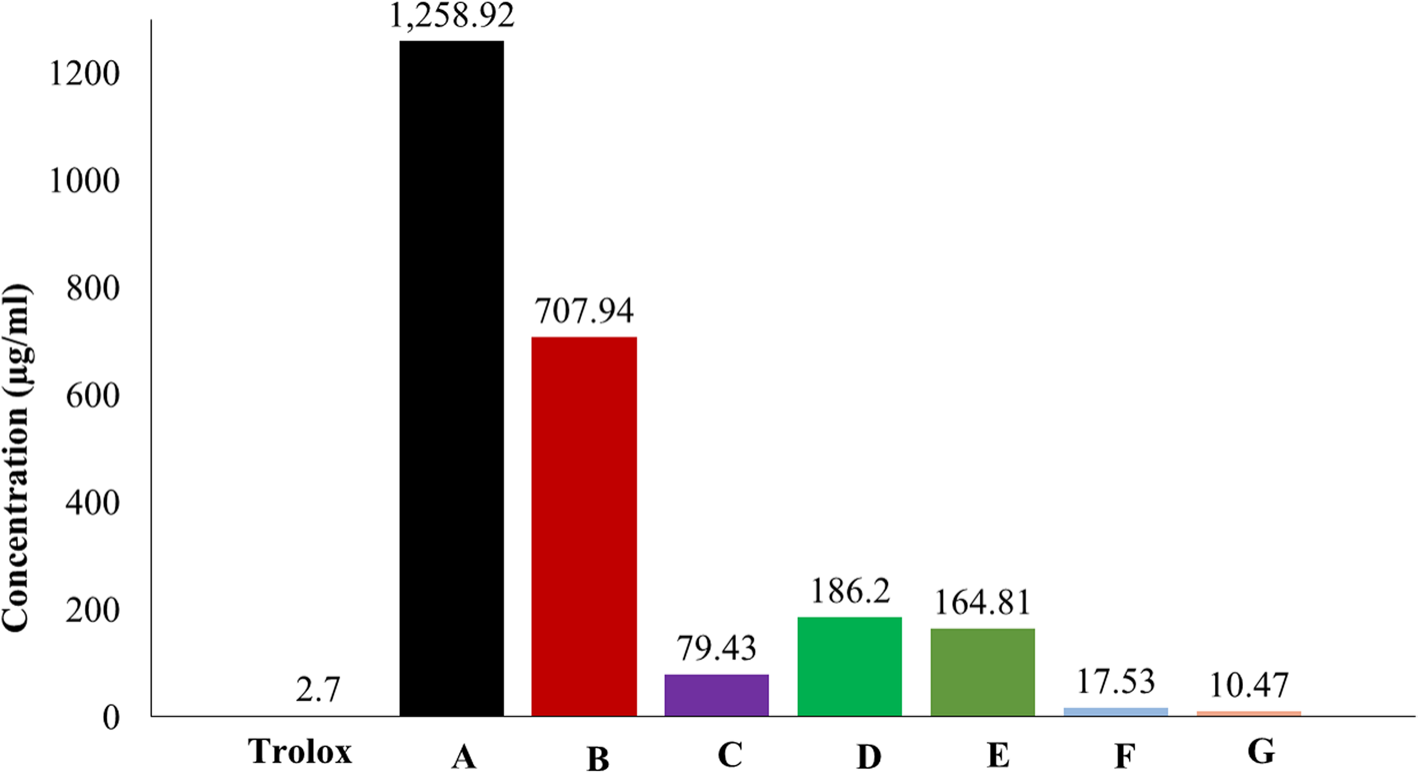
IC_50_ values in (μg/ml) for trolox and the tested products

The antioxidant effect for all tested products was obtained using the DPPH method. The results showed that the seven products had different antioxidant activity when compared to trolox, as shown in Fig. 5. The IC_50_ values for the seven products (A®, B®, C®, D®, E®, F®, and G®) were 1258.92, 707.94, 79.43, 186.20, 164.81, 17.53, and 10.47 μg/ml respectively, while the IC_50_ value for trolox was 2.70 μg/ml (Fig. 6). The strongest antioxidant effect was with product G®, and the lowest was with product A®.

## Discussion

Obesity is a serious global health problem and should be regarded as a worldwide pandemic. Few botanicals contained in natural weight reduction solutions have been subjected to extensive fundamental and clinical study, and it is crucial that this be done to verify their broad use for weight control. Many natural weight-loss products are sold and used around the world with no (or little) evidence of their effectiveness or quality [44]. Therefore, this study was conducted to evaluate and investigate the effectiveness of some herbal products certified and presented in the Palestinian community that are used to treat obesity. The results obtained were compared with those of commercial obesity medications that exert their beneficial effect in the same pathway. They were tested for lipase, amylase, and oxidation inhibition, then the IC_50_ value was calculated to compare the results with the reference products.

Pancreatic lipase is one of the most important enzymes for the digestion of dietary triacylglycerols [45]. It is known that dietary lipids are not directly absorbed from the intestines unless they have been subjected to the action of pancreatic lipase. It has been clinically noticed that the pancreatic lipase inhibitor orlistat has a beneficial approved effect on obesity and hyperlipidemia by increasing fat excretion through the feces and by inhibiting pancreatic lipase [46].

According to the results obtained through this research, product F® showed the highest inhibition potency on lipase, which was composed of amino acids, vitamin B6, chromium, and *taraxacum officinale* extract. The effect of this drug when it comes to pancreatic lipase inhibition is believed to be related to the *taraxacum officinale* extract, which was previously studied in vivo and in vitro*,* showing potent inhibition of lipase. A recent study by Aabideen et al. [31] found that this plant has hypolipidemic activity mostly via inhibition of pancreatic lipase [31]. Another study by Jian Zhag et al. showed the same good activity of the *T. officinale* extract compared to the orlistat drug [32]. In addition to that, product G® showed the next best result, which was composed of green tea extract and phytosome complex. It was reported that a green tea extract significantly inhibited gastric and pancreatic lipase activities, as determined by using a relatively high level of catechins under gastric and duodenal conditions in vitro [47]. A similar finding was reported by Christine Juhel et al, [48] who clearly showed the activity of green tea extract in the inhibition of lipase, which could be useful in reducing body weight in obese people [48].

α-Amylase is a digestive enzyme secreted by the pancreas and salivary glands that aids in carbohydrate digestion. α-Amylase inhibitors work by slowing carbohydrate digestion and absorbing postprandial glucose to reduce postprandial hyperglycemia. Reducing postprandial hyperglycemia will stop glucose uptake into adipose tissue, which will inhibit the synthesis and accumulation of triacylglycerol. The inhibition of these digestive enzymes has an important role in the treatment of obesity by reducing weight gain in obese people [49]. According to the findings, F® had the most effective herbal product for amylase inhibition. This result may be related to amino acids and vitamin B6, which are among its ingredients. Another study was conducted by Kim et al. [50] to investigate the inhibitory effect of pyridoxine against some digestive enzymes, including a-glucoamylase, which showed a decrease in the minimum, maximum, and mean levels of postprandial blood glucose at 0.5 hour after meals [50]. Product G® has the next best value and contains green tea extract. They have been shown to be beneficial in the treatment of type 2 diabetes, and one possible mechanism is their inhibitory effect on α -amylase and -glucosidase in the digestive tract. A similar study was conducted by Ming Miao et al. to indicate the effectiveness of green tea extract in inhibiting the a-amylase enzyme, which is bound together to form a complex with a static quenching mechanism [51]. Also, product D® showed a high anti-amylase effect as it is composed of *Garcinia Cambogia* fruit extract, an endemic evergreen tree that can be found in Taiwan. Chen et al. previously investigated the ability of the same extract to inhibit the same enzyme activity and delay carbohydrate digestion [52]. Product E®, which contains *hoodia gordonii* extract, showed good results as well. However, product A® showed a low anti-amylase effect.

Animal and clinical studies have reported the role of oxidative stress in weight gain pathogenesis, which can trigger obesity by stimulating the deposition of adipose tissue. Antioxidant therapy was found to help alter body weight and reduce the possible incidence of metabolic diseases [53]. High amounts of circulating lipids and glucose may result in an oversupply of energy substrates for metabolic pathways in several human cells, including adipocytes, which can increase the formation of reactive oxygen species. Obesity and its associated diseases are thus linked to oxidative stress [54]. Several studies have found that in vitro gastrointestinal digestion contributes to structure alteration and antioxidant activity [55]. Antioxidants produce this weight loss effect by effectively reducing enzymes responsible for fat buildup in the cells as well as destroying fat cells, thus reducing the accumulation of fats in the body. Fats enriched with fiber can also stop weight gain by stimulating the elimination of waste and giving the feeling of satiety for longer periods of time, they can also help burn fat more efficiently [56]. Furthermore, the best antioxidant effect was shown with products F® and G®. It is believed to be due to their separate compositions. Close to that comes product C®, containing *Laurus nobilis, Cymbopogon citratus*, and vitamin C. Product E® contains *Hoodia gordonii* extract, as described in another article by Petrine Kapewangolo et al. *H. gordonii* extract demonstrated a good antioxidant activity, its extracts reducing power increased as concentration increased, indicating that the extracts included antioxidants (reductants) (phenolics, alkaloids, terpenes, steroids, cardiac glycosides, and tannins) [57]. Next came products E® and D®, which contain *Garcinia Cambogia* fruit extract [58], and finally, product B® (low antioxidant effect) contains vitamin E. The products chosen for this study contained a variety of natural materials. These natural materials have been scientifically proven to have activity in the management of obesity. However, based on the findings of this research, any natural product needs to be verified scientifically, as it was proved in this study that some of these products do not have any contribution to obesity management.

### Strengths and limitations

The main strength of this study is that it was the first to be conducted in Palestine to evaluate the efficacy and quality of weight-loss herbal products used in the Palestinian community. Furthermore, this study will serve as a guideline for pharmaceutical companies when dealing with herbal products. One of the limitations of our study was that the name of the product could not be revealed in order to maintain privacy.

## Conclusion

In conclusion, results of this study showed that product F®, which contains amino acids, vitamin B6, chromium, and *taraxacum officinale* extract, had the highest anti-lipase and anti-amylase effects when compared with the seven herbal formulations. In addition, we found that product G®, which contains green tea extracts and a phytosome complex, had the highest antioxidant activity. In addition, the study reveals that some herbal products available in the Palestinian market do not have any valuable potency in the management of obesity. Therefore, investigations into the potency of any herbal product must be conducted before it is allowed on the market. This issue was one of the main goals of this study, in order to raise patient and pharmacist awareness regarding this matter and to ensure that patients get the best treatment possible. Moreover, future studies must be conducted on the safety of these products.

## Data Availability

The datasets used and/or analysed during the current study are available from the corresponding author on reasonable request.

## References

[1] Guran T, Bereket A (2011). International epidemic of childhood obesity and television viewing. Minerva Pediatr.

[2] Hilton S, Patterson C, Teyhan A (2012). Escalating coverage of obesity in UK newspapers: the evolution and framing of the “obesity epidemic” from 1996 to 2010. Obesity.

[3] Sumińska M, Podgórski R, Bogusz‐Górna K, Skowrońska B, Mazur A, Fichna M. Historical and cultural aspects of obesity: from a symbol of wealth and prosperity to the epidemic of the 21st century. Obes Rev. 2022;24:e13440. 10.1111/obr.13440 35238142

[4] Mafort TT, Rufino R, Costa CH, Lopes AJ (2016). Obesity: systemic and pulmonary complications, biochemical abnormalities, and impairment of lung function. Multidiscip Respir Med.

[5] Collaborators GO (2017). Health effects of overweight and obesity in 195 countries over 25 years. N Engl J Med.

[6] Anderson E, Durstine JL (2019). Physical activity, exercise, and chronic diseases: a brief review. Med Sci Sports Exerc.

[7] Allison DB, Gadde KM, Garvey WT, Peterson CA, Schwiers ML, Najarian T, Tam PY, Troupin B, Day WW (2012). Controlled-release phentermine/topiramate in severely obese adults: a randomized controlled trial (EQUIP). Obesity.

[8] Daudén E, Castañeda S, Suárez C, García-Campayo J, Blasco A, Aguilar M, Ferrándiz C, Puig L, Sánchez-Carazo J (2013). Psoriasis WGoCi: clinical practice guideline for an integrated approach to comorbidity in patients with psoriasis. J Eur Acad Dermatol Venereol.

[9] Meyre D, Boutin P, Froguel P (2004). Obesity: genetics, behavior or a combination of both. Curr MedChem Immunol Endoc Metab Agents.

[10] Shubayr MA, Mattoo KA (2020). Parental neglect of feeding in obese individuals: a review of scientific evidence and its application among Saudi population. Saudi Med J.

[11] Roberto CA, Kawachi I (2014). Use of psychology and behavioral economics to promote healthy eating. Am J Prev Med.

[12] Sarma S, Sockalingam S, Dash S (2021). Obesity as a multisystem disease: trends in obesity rates and obesity-related complications. Diabetes Obes Metab.

[13] Safaei M, Sundararajan EA, Driss M, Boulila W, Shapi'i A (2021). A systematic literature review on obesity: understanding the causes & consequences of obesity and reviewing various machine learning approaches used to predict obesity. Comput Biol Med.

[14] Rubino F, Cohen RV, Mingrone G, le Roux CW, Mechanick JI, Arterburn DE, Vidal J, Alberti G, Amiel SA, Batterham RL (2020). Bariatric and metabolic surgery during and after the COVID-19 pandemic: DSS recommendations for management of surgical candidates and postoperative patients and prioritisation of access to surgery. Lancet Diabetes Endocrinol.

[15] Braidy N, Villalva MD (2020). Eeden Sv: sobriety and satiety: is NAD+ the answer?. Antioxidants.

[16] Bosello O, Vanzo A (2021). Obesity paradox and aging. Eat Weight Disord-St.

[17] Dietz WH, Baur LA, Hall K, Puhl RM, Taveras EM, Uauy R, Kopelman P (2015). Management of obesity: improvement of health-care training and systems for prevention and care. Lancet.

[18] Burgess E, Hassmén P, Pumpa KL (2017). Determinants of adherence to lifestyle intervention in adults with obesity: a systematic review. Clin Obes.

[19] Shende P, Narvenker R (2021). Herbal nanotherapy: a new paradigm over conventional obesity treatment. J Drug Deliv Sci Technol.

[20] Balaji M, Ganjayi MS, Kumar GEH, Parim BN, Mopuri R, Dasari S (2016). A review on possible therapeutic targets to contain obesity: the role of phytochemicals. Obes Res Clin Pract.

[21] Gurevich-Panigrahi T, Panigrahi S, Wiechec E, Los M (2009). Obesity: pathophysiology and clinical management. Curr Med Chem.

[22] Patel DK, Stanford FC (2018). Safety and tolerability of new-generation anti-obesity medications: a narrative review. Postgrad Med.

[23] Yang H, Yue GGL, Leung PC, Wong CK, San Lau CB. A review on the molecular mechanisms, the therapeutic treatment including the potential of herbs and natural products, and target prediction of obesity-associated colorectal cancer. Pharmacol Res. 2021:106031. 10.1016/j.phrs.2021.106031 34896542

[24] Negi H, Gupta M, Walia R, Khataibeh M, Sarwat M (2021). Medicinal plants and natural products: more effective and safer pharmacological treatment for the Management of Obesity. Curr Drug Metab.

[25] Payab M, Hasani-Ranjbar S, Shahbal N, Qorbani M, Aletaha A, Haghi-Aminjan H, Soltani A, Khatami F, Nikfar S, Hassani S (2020). Effect of the herbal medicines in obesity and metabolic syndrome: a systematic review and meta-analysis of clinical trials. Phytother Res.

[26] Sun N-N, Wu T-Y, Chau C-F (2016). Natural dietary and herbal products in anti-obesity treatment. Molecules.

[27] Xu Y, Zhang M, Wu T, Dai S, Xu J, Zhou Z (2015). The anti-obesity effect of green tea polysaccharides, polyphenols and caffeine in rats fed with a high-fat diet. Food Funct.

[28] Janssens PL, Hursel R, Westerterp-Plantenga MS (2016). Nutraceuticals for body-weight management: the role of green tea catechins. Physiol Behav.

[29] Westerterp-Plantenga M (2010). Green tea catechins, caffeine and body-weight regulation. Physiol Behav.

[30] Abunofal O, Mohan C (2022). Salubrious effects of green tea Catechins on fatty liver disease: a systematic review. Medicines.

[31] Aabideen ZU, Mumtaz MW, Akhtar MT, Mukhtar H, Raza SA, Touqeer T, Saari N (2020). Anti-obesity attributes; UHPLC-QTOF-MS/MS-based metabolite profiling and molecular docking insights of Taraxacum officinale. Molecules.

[32] Zhang J, Kang M-J, Kim M-J, Kim M-E, Song J-H, Lee Y-M, Kim J-I (2008). Pancreatic lipase inhibitory activity of taraxacum officinale in vitro and in vivo. Nutr Res Pract.

[33] Mahboubi M, Mahboubi M (2020). Hepatoprotection by dandelion (Taraxacum officinale) and mechanisms. Asian Pac J Trop Biomed.

[34] García-Carrasco B, Fernandez-Dacosta R, Dávalos A, Ordovás JM, Rodriguez-Casado A (2015). In vitro hypolipidemic and antioxidant effects of leaf and root extracts of Taraxacum officinale. Med Sci.

[35] Lis B, Olas B (2019). Pro-health activity of dandelion (Taraxacum officinale L.) and its food products–history and present. J Funct Foods.

[36] Tiikkainen M, Bergholm R, Rissanen A, Aro A, Salminen I, Tamminen M, Teramo K, Yki-Järvinen H (2004). Effects of equal weight loss with orlistat and placebo on body fat and serum fatty acid composition and insulin resistance in obese women. Am J Clin Nutr.

[37] Kaefer CM, Milner JA (2008). The role of herbs and spices in cancer prevention. J Nutr Biochem.

[38] Oduola T, Bello I, Adeosun G, Ademosun A-W, Raheem G, Avwioro G (2010). Hepatotoxicity and nephrotoxicity evaluation in Wistar albino rats exposed to Morinda lucida leaf extract. N Am J Med Sci.

[39] Krishnaraju A, Sundararaju D, Srinivas P, Rao C, Sengupta K, Trimurtulu G (2010). Safety and toxicological evaluation of a novel anti-obesity formulation LI85008F in animals. Toxicol Mech Methods.

[40] Hamidpour R, Rashan L (2017). An herbal preparation useful in weight loss. Transl Biomed.

[41] Jaradat N, Khasati A, Hawi M, Hawash M, Shekfeh S, Qneibi M, Eid AM, Arar M, Qaoud MT (2022). Antidiabetic, antioxidant, and anti-obesity effects of phenylthio-ethyl benzoate derivatives, and molecular docking study regarding α-amylase enzyme. Sci Rep.

[42] Eid AM, Hawash M (2021). Biological evaluation of Safrole oil and Safrole oil Nanoemulgel as antioxidant, antidiabetic, antibacterial, antifungal and anticancer. BMC Complement Med Ther.

[43] Jaradat N, Abualhasan M (2016). Comparison of phytoconstituents, total phenol contents and free radical scavenging capacities between four Arum species from Jerusalem and Bethlehem. Pharm Sci.

[44] Arya A, Nahar L, Khan HU, Sarker SD, Sarker SD, Nahar L (2020). Chapter thirteen - anti-obesity natural products. Annual reports in medicinal chemistry.

[45] Wilcox MD, Brownlee IA, Richardson JC, Dettmar PW, Pearson JP (2014). The modulation of pancreatic lipase activity by alginates. Food Chem.

[46] Jabir AS, Iraby AG (2014). Studying the effect of anti-amylase inhibitor extracted from white kidney bean (Phaseolus vulgaris) in treat diabetes and obesity in an affected mice. Int J Curr Microbiol App Sci.

[47] Koo SI, Noh SK (2007). Green tea as inhibitor of the intestinal absorption of lipids: potential mechanism for its lipid-lowering effect. J Nutr Biochem.

[48] Juhel C, Armand M, Pafumi Y, Rosier C, Vandermander J, Lairon D (2000). Green tea extract (AR25®) inhibits lipolysis of triglycerides in gastric and duodenal medium in vitro. J Nutr Biochem.

[49] Rani N, Vasudeva N, Sharma SK (2012). Quality assessment and anti-obesity activity of Stellaria media (Linn.) Vill. BMC Complement Med Ther..

[50] Kim HH, Kang Y-R, Lee J-Y, Chang H-B, Lee KW, Apostolidis E, Kwon Y-I (2018). The postprandial anti-hyperglycemic effect of pyridoxine and its derivatives using in vitro and in vivo animal models. Nutrients.

[51] Miao M, Jiang B, Jiang H, Zhang T, Li X (2015). Interaction mechanism between green tea extract and human α-amylase for reducing starch digestion. Food Chem.

[52] Chen T-H, Fu Y-S, Chen S-P, Fuh Y-M, Chang C, Weng C-F (2021). Garcinia linii extracts exert the mediation of anti-diabetic molecular targets on anti-hyperglycemia. Biomed Pharmacother.

[53] Manna P, Jain SK (2015). Obesity, oxidative stress, adipose tissue dysfunction, and the associated health risks: causes and therapeutic strategies. Metab Syndr Relat Disord.

[54] Podsędek A, Majewska I, Kucharska AZ (2017). Inhibitory potential of red cabbage against digestive enzymes linked to obesity and type 2 diabetes. J Agric Food Chem.

[55] Chen W, Su H, Xu Y, Bao T, Zheng X (2016). Protective effect of wild raspberry (Rubus hirsutus Thunb.) extract against acrylamide-induced oxidative damage is potentiated after simulated gastrointestinal digestion. Food Chem.

[56] Fernández-Sánchez A, Madrigal-Santillán E, Bautista M, Esquivel-Soto J, Morales-González Á, Esquivel-Chirino C, Durante-Montiel I, Sánchez-Rivera G, Valadez-Vega C, Morales-González JA (2011). Inflammation, oxidative stress, and obesity. Int J Mol Sci.

[57] Kapewangolo P, Knott M, Shithigona RE, Uusiku SL, Kandawa-Schulz M (2016). In vitro anti-HIV and antioxidant activity of Hoodia gordonii (Apocynaceae), a commercial plant product. BMC Complement Med Ther.

[58] Suk S, Kwon GT, Lee E, Jang WJ, Yang H, Kim JH, Thimmegowda N, Chung MY, Kwon JY, Yang S (2017). Gingerenone a, a polyphenol present in ginger, suppresses obesity and adipose tissue inflammation in high-fat diet-fed mice. Mol Nutr Food Res.

